# Supplementary material for: Belief Inflexibility and Cognitive Biases in Anorexia Nervosa—The Role of the Bias against Disconfirmatory Evidence and Its Clinical and Neuropsychological Correlates
Source: J Clin Med. 2023 Feb 22;12(5):1746. doi: 10.3390/jcm12051746 (PMC10003469; doi:10.3390/jcm12051746)
Supplement: Supplementary file 1 [file jcm-12-01746-s001.zip › jcm-2216604-supplementary.pdf]

Supplemental Materials

Analyses of plausibility rates (Figure S1-) showed that the difficulties displayed by patients with AN in integrating evidence is qualitatively different from the dysfunctional pattern observed in schizophrenia individuals. While in schizophrenia the differences between patients and controls increases in levels 2 and 3, due to the inability of patients to change their plausibility rating, in AN the differences occurred especially in the first two levels, when little information is available and high levels of uncertainty are still present.

Figure S1: Changes in plausibility rating during the task in health women and acute AN for the four different types of scenarios

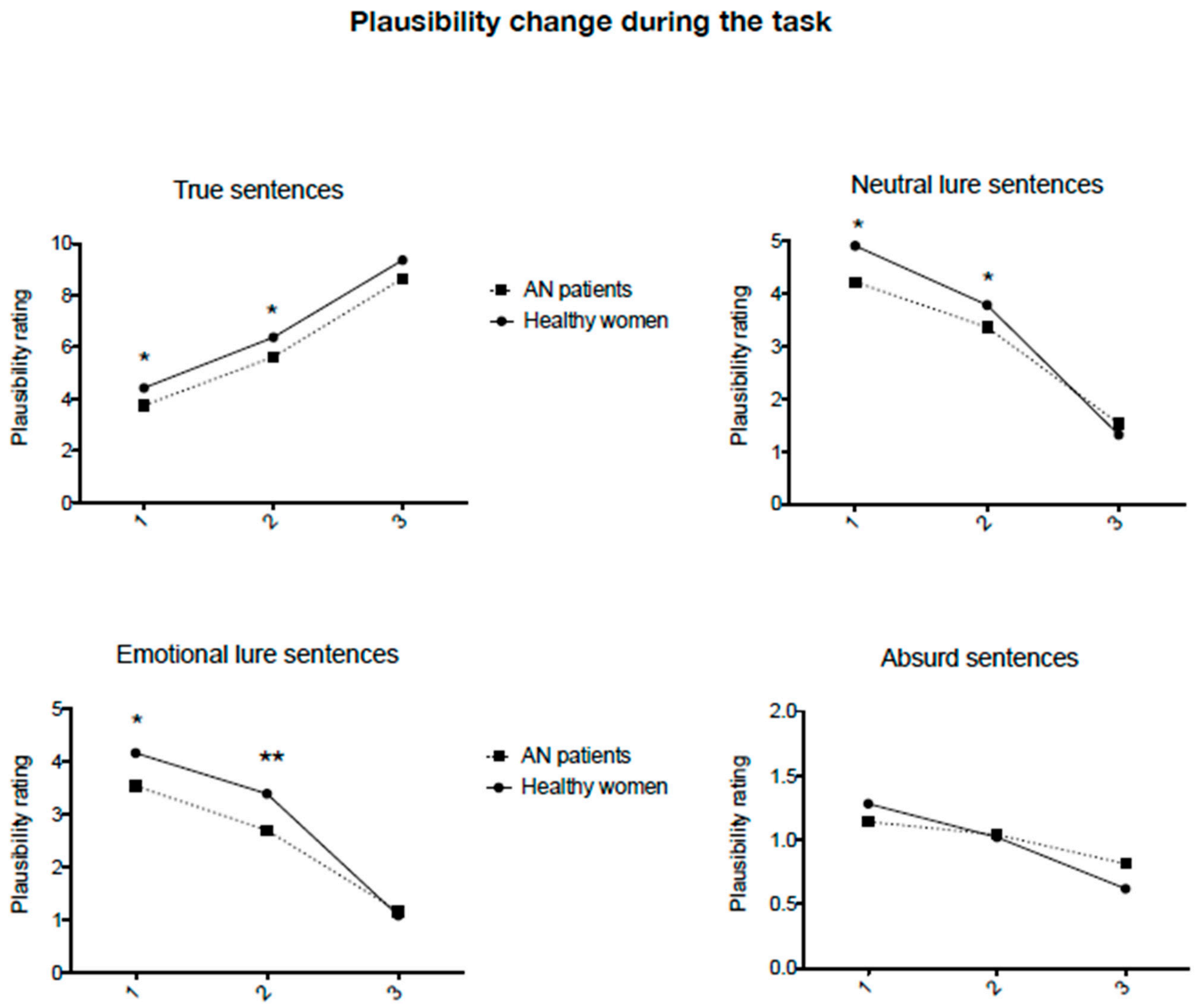

Note: 1, 2, 3: first, second and third statements progressively adding information to the scenarios as the task progresses; Kruskal-Wallis test; * p<0.05; ** p<0.01
